# Alterations in Gut Microbiome-Host Relationships After Immune Perturbation in Patients With Multiple Sclerosis

**DOI:** 10.1212/NXI.0000000000200355

**Published:** 2025-01-16

**Authors:** Vinod K. Gupta, Guneet S. Janda, Heather K. Pump, Nikhil Lele, Isabella Cruz, Inessa Cohen, William E. Ruff, David A. Hafler, Jaeyun Sung, Erin E. Longbrake

**Affiliations:** 1 Microbiomics Program, Center for Individualized Medicine, Mayo Clinic, Rochester, MN;; 2 Yale School of Medicine Department of Neurology, New Haven, CT;; 3 Mayo Clinic Alix School of Medicine, Mayo Clinic, Rochester, MN;; 4 Yale School of Medicine Department of Immunobiology, New Haven, CT;; 5 Division of Computational Biology, Department of Quantitative Health Sciences, Mayo Clinic; and; 6 Division of Rheumatology, Department of Medicine, Mayo Clinic, Rochester, MN.

## Abstract

**Background and Objectives:**

Gut microbial symbionts have been shown to influence the development of autoimmunity in multiple sclerosis (MS). Emerging research points to an important relationship between the microbial-IgA interface and MS pathophysiology. IgA-secreting B cells are observed in the MS brain, and shifts in gut bacteria-IgA binding have been described in some patients with MS. However, the relationships between the gut microbiome and the host immune response, particularly regarding B-cell–depleting immunomodulation, remain underexplored. This study aimed to evaluate the composition of the gut microbiome in patients with newly diagnosed MS at baseline and after B-cell depletion, using long-read sequencing for enhanced taxonomic resolution. We further aimed to investigate the host/microbiome interface by evaluating microbe/immunoglobulin A relationships.

**Methods:**

We collected stool samples from 43 patients with newly diagnosed, untreated MS and 42 matched healthy controls. Nineteen patients with MS initiated anti-CD20 monoclonal antibody treatment and donated additional stool samples after 6 months of treatment. We evaluated the host-microbial interface using bacterial flow cytometry and long-read 16S rRNA gene amplicon sequencing. We used Immune Coating Scores to compare the proportions of bacteria identified in the IgA-coated vs IgA-uncoated bacterial fractions.

**Results:**

Patients with untreated, newly diagnosed MS showed significant reductions in IgA-bound fecal microbiota compared with controls. Using multiple linear regression models adjusted for potential confounders, we observed significant (*p* < 0.05) changes in the abundance and prevalence of various strain-level gut bacteria amplicon sequence variants (ASVs) within both total and IgA-coated bacterial fractions. Some changes (e.g., decreased relative abundance of a *Faecalibacterium prausnitzii* variant in MS) were consistent with previous reports, while others (e.g., increased relative abundance and prevalence of *Monoglobus pectinyliticus* in MS) were novel. Immune Coating Scores identified subsets of organisms for which normal IgA-coating patterns were disrupted at the onset of MS, as well as those (particularly *Akkermansia muciniphila*) whose IgA-coating became more aligned with controls after therapy.

**Discussion:**

This analysis of gut microbial ASVs reveals shifts in taxonomic strains induced by immune modulation in MS.

## Introduction

Gut microbial symbionts occupy a liminal space that bridges the internal host environment with external factors. Alterations in the taxonomic and functional composition of this gut microbiome have been implicated as significant contributors to autoimmune diseases, such as multiple sclerosis (MS). Recent evidence supports this link, showing that certain T-cell clones within MS lesions recognize guanosine diphosphate (GDP) l-fucose synthase, a human autoantigen with a conserved bacterial homolog.^1^ The study also found that these CNS T cells cross-reacted with GDP l-fucose synthase and gut microbiota. In another study, epsilon toxin-producing *Clostridium perfringens* species were more abundant in patients with MS. This toxin proved to be an effective adjuvant for inducing experimental autoimmune encephalitis.^2^ Further supporting the role of the gut microbiome in autoimmunity, mechanistic studies have shown that fecal microbial transfer from patients with MS induces a higher incidence of spontaneous brain autoimmune disease in a transgenic mouse model compared with fecal transfer from unaffected twins.^3^ Observational studies in humans have reported different gut microbiome profiles in patients with MS compared with healthy individuals.^4-9^ However, the functional significance of these differences remains unclear. This knowledge gap may be partly due to previous research focusing primarily on gut microbial taxonomies, and thus overlooking the complex, dynamic relationship between the host and the microbiome. Indeed, microbial contributions to disease may not lie in specific taxa but in the broader ecological dynamics and the intricate host-microbe interface.

Despite the wealth of research on microbial composition changes in MS, the host's response to its gut microbiome, particularly the interaction between host immunoglobulin A (IgA) and commensal organisms, is not well understood. Secreted IgA plays a critical role in regulating microbial composition along the gastrointestinal tract^10,11^ by opsonizing mucosal and luminal organisms, limiting their motility, and targeting them for phagocytosis or sequestration.^12^ IgA binding can also modulate bacterial gene expression in ways that benefit the host.^12,13^ Typically, pathobionts trigger a strong IgA-coating response, except in individuals with IgA deficiency.^10^ In IgA-deficient individuals, differences in gut microbial abundance and increased susceptibility to autoimmune or inflammatory conditions (including celiac disease, type 1 diabetes, and inflammatory bowel disease) have been observed.^14-16^

Emerging research points to an important relationship between the microbial-IgA interface and MS pathophysiology. It has been observed that IgA-secreting B cells that target gut microbes migrate from the gut to the CNS during inflammatory disease states, where they adopt a regulatory phenotype.^17,18^ By contrast, we previously found that proinflammatory T cells from patients with MS disproportionately express gut-homing receptors.^19^ Despite these recent advancements, the specific characteristics of the host-microbe interface at MS onset, as well as the effect of immunomodulatory disease-modifying therapies on this interaction, have yet to be closely examined.

In this study, we investigate the effects of B-cell depletion therapy on the host-microbe interface in MS. We examine the interactions between host IgA and gut microbiota in newly diagnosed patients with MS who had not yet undergone any disease-modifying, immunomodulatory treatments. We then explore how anti-CD20 B-cell–depleting therapies affect these interactions over time. Our findings reveal a significant disruption in the host-microbe interface at the onset of clinical disease, particularly in the engagement between microbial antigens and IgA. Following B-cell–depleting therapy, IgA-coating patterns began to realign with those seen in healthy individuals.

## Methods

### Sex as a Biological Variable

Both male and female participants were included in this study, and all statistical models were adjusted for sex where applicable.

### Study Design

This observational cohort study had both cross-sectional and longitudinal components. Patients with newly diagnosed, treatment naïve MS were sequentially recruited from the Yale Multiple Sclerosis Center clinic population. Age-matched and sex-matched healthy individuals were also recruited as controls. Inclusion criteria for patients included a new diagnosis of MS (within the past year) and willingness to provide stool samples. Participants were excluded if they had any prior long-term exposure to immunomodulatory medications (e.g., any disease-modifying therapy), antibiotic use within 3 months of sample collection, or preexisting diagnoses of irritable bowel syndrome or small intestinal bacterial overgrowth.

All participants completed questionnaires regarding current and past diet, medications, and exposures, along with the Automated Self-Administered 24-hour Dietary Assessment Tool (ASA24), which covered the 24-hour period before stool sample collection. Stool samples were collected by participants at home, immediately frozen, and shipped overnight to the laboratory, where they were aliquoted and stored at −80°C. All samples were collected at Yale University between 2018 and 2021.

**Figure 1:**
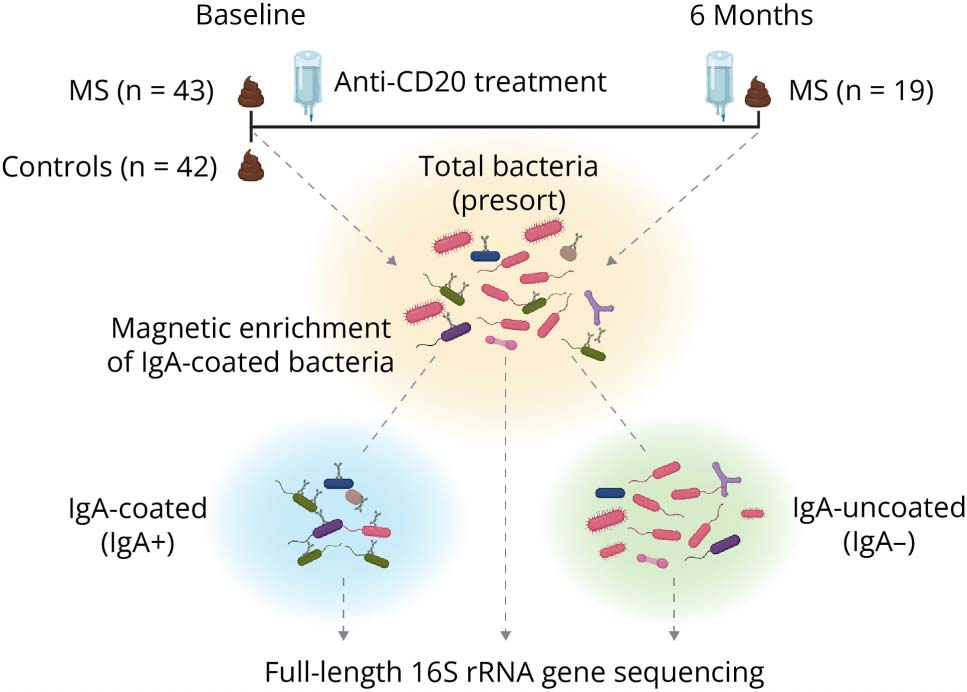
Overview of Study Design This was an observational study incorporating cross-sectional and longitudinal components. Baseline stool samples were collected from 43 patients with treatment-naïve MS and 42 matched healthy controls. Longitudinal samples were acquired 6 months after beginning B-cell–depleting therapy for n = 19 patients with MS. All samples underwent IgA-based sorting and full-length 16S rRNA gene amplicon sequencing to assess the gut microbiome and to compare the total (presort), IgA-coated (IgA+), and IgA-uncoated (IgA–) bacterial fractions before and after treatment. Figure created with Biorender. IgA = immunoglobulin A; MS = multiple sclerosis.

The study design is illustrated in Figure 1. All participants donated blood and stool samples at baseline, with a subset of patients with MS providing additional samples 6 months after initiating B-cell–depleting immunotherapies (anti-CD20 monoclonal antibodies). These therapies are highly effective in controlling inflammatory disease activity^20^ and are known to rapidly induce lysis of mature B cells, which typically repopulate over a median of approximately 12 months.^21^ In clinical practice, anti-CD20 medications are dosed every 6 months to maintain complete depletion of circulating B cells. A planned sample size of 40 participants per group was determined based on preliminary data for the effect size and SD of specific organisms of interest.

### Bac-FACS and IgA-Seq

Fecal samples were diluted, homogenized, and pelleted to remove insoluble factors. The supernatants were further centrifuged to pellet the bacteria, which were then blocked and labeled with PE-conjugated IgA, IgG, and IgM (Miltenyi, IS11-8E10; Jackson Immuno Research 109-605-098 and 709-096-073, respectively). The fecal microbiome was analyzed in total and based on immunoglobulin coating (e.g., percent coated) using flow cytometry (BD LSRFortessa) before and after magnetic enrichment of IgA-coated fractions (StemCell Technologies 17654). The estimated number of bacteria per sample using this technique exceeds 1 × 10^7^.^22^ Soluble, unbound IgA in the fecal supernatants was quantified using an ELISA kit (Eagle Biosciences, SGA35-K01).

*DNA isolation and long-read amplicon sequencing:* DNA from total (presort), IgA+, and IgA– bacterial fractions was isolated and amplified using the Shoreline Complete Strain ID Lyse, Purify & Amplify kit. This kit amplifies long-read amplicons encompassing the 16S-ITS-23S rRNA gene regions. Subsequently, sequencing was performed using the PacBio system.

*Taxonomic profiling of full-length 16s rRNA gene reads:* SBanalyzer 3.0 (Shoreline Biome) was used to demultiplex and classify CCS.fastq reads from the Sequel II sequencing run through the StrainID_PacBio_species pipeline, leveraging the Athena database for taxonomic classification. The Athena database, an integrated component of the SBanalyzer pipeline, contains contiguous 16S-23S sequences from 19,185 genomes, including 5,551 “reference” and “representative” genomes and 13,634 other genomes assembled at the “complete” and “chromosome” levels (downloaded from RefSeq on May 21, 2019).

Further analysis was conducted using DADA2 in R to detect sequencing errors and group reads into amplicon sequence variants (ASVs) for taxonomic assignment.^23^ The DADA2 R package implements a comprehensive workflow that processes raw amplicon sequencing data and provides an error-corrected table of the abundances of ASVs in each sample. Standard steps in the DADA2 workflow include quality filtering, dereplication, learning the error model specific to the data set, variant inference, chimera removal, and taxonomic assignment.

To determine the relative abundances of strain-level ASVs in each sample, the observed frequency of ASVs for a given taxonomic strain was divided by the total number of sequences in that sample (sequencing depth). Only samples with an ASV count exceeding 2,474 were considered for further analysis (eFigure 1).

### IgA-Coating Score

To identify bacterial strains preferentially present in the IgA + fractions compared with the IgA– fractions, an IgA-Coating Score (ICS) for each strain-level ASV was calculated using the following formula:

$$ICS=\log_{2}\left(\frac{p_{m,I_{g}A+}+c}{p_{m,I_{g}A–}+c}\right)$$

where $p_{m,I_{g}A+}$ and $p_{m,I_{g}A–}$ represent the prevalence of microbe m (i.e., proportion of participants who have microbe m) in the IgA+ and IgA– fractions, respectively. The variable c is a pseudocount added to avoid division by zero and is set at 1.0 × 10^−5^.

Prevalence was chosen over relative abundance to define the ICS, primarily due to the inherent sparsity of microbiome data. Prevalence, which is based on a microbe's presence or absence across a sample set, is less susceptible to the distortions that can affect relative abundance measurements, which are influenced by the absolute number of organisms. It also avoids the misleading effects that can arise from averaging relative abundance levels or creating ratios. Prevalence provides a binary, yet informative, perspective on the association of microbes with specific conditions, highlighting consistent presence rather than quantity. This approach is crucial for identifying microbes that despite their lower abundance, hold potential biological significance that would otherwise be overlooked.

Only strains present in at least 10% of all study samples were included in the analysis to avoid skewing results due to rare organisms. *p* values for ICSs were calculated using a class-label permutation test, where the null hypothesis asserts no difference in prevalence between IgA+ and IgA– fractions (i.e., ICS = 0). The threshold for statistical significance was set to *p* < 0.05, indicating that an ASV had a significantly positive ICS within a study group. ASVs with significantly negative ICS scores were not considered in the analysis.

### Statistical Analysis

The Shannon α-diversity (Shannon Index) was calculated for each sample using the R package “vegan” (v2.6-4) based on ASV counts. Multiple linear regression models (MLRMs) were constructed in R (v4.0.3) to assess the differences in microbial α-diversity between controls and patients with untreated MS, with significance set at *p* < 0.05. The *p* value for the α-diversity coefficient in the MLRM, after adjusting for potential confounders, indicated statistical significance.

Bray-Curtis distance matrices were generated based on arcsine, square-root transformed relative abundances of ASVs between stool samples for each bacterial fraction (presort, IgA+, and IgA–). These matrices were generated using R packages “ade4” (v1.7-22) and “vegan” (v2.6-4). A Permutational Multivariate Analysis of Variance was conducted on the matrices with the “adonis2” function in R (v4.0.3) to determine the extent to which the study group contributed to the overall variation in gut microbiome composition. This analysis accounted for age, sex, body mass index (BMI), and steroid use as potential confounders, using 999 permutations to determine the *p* value and R^2^.

The compositional nature of microbiome data was accounted for by applying a centered log-ratio (clr) transformation to the relative abundances following the addition of a small pseudocount (1.2 × 10^−5^) to zeros. MLRMs were then applied to ASVs (with at least 10% prevalence in each bacterial fraction) to identify differentially abundant ASVs between study groups. These models were adjusted for confounders (i.e., age, sex, BMI, and steroid use) with *p* < 0.05 indicating statistical significance. Finally, mixed-effects linear regression models, adjusted for steroid use and intrasubject longitudinal variation, were used to identify differentially abundant ASVs between paired samples from patients with MS at baseline and 6-month posttreatment.

### Study Approval

This study was approved by the Yale Institutional Review Board. All study participants provided written informed consent before participation. Data reporting is consistent with Strengthening the Reporting of Observational Studies in Epidemiology guidelines.

### Data Availability

Raw sequencing data for the 16s rRNA gene amplicons used in this study have been deposited in NCBI's Sequence Read Archive data repository under the BioProject number PRJNA1099675. The deposited sequences comprise 289 total “.fastq” files for stool samples, which were collected from 43 patients with MS and 42 controls.

## Results

### Study Design

Stool samples were collected from 43 patients with newly diagnosed, untreated MS and 42 age-matched and sex-matched healthy (no disease) individuals as controls (Table). Most participants exhibited mild to moderate neurologic disability at enrollment (EDSS of ≤3 for 81% of participants). Sample collections took place an average of 1.6 months after diagnosis, before initiation of disease-modifying therapy. About half of the participants had experienced a relapse within 3 months of enrollment, and a similar proportion had received steroids as treatment for a relapse. There were no significant differences in baseline dietary intake between patients and controls (eTable 1). Nineteen patients with MS initiated B-cell–depleting immunotherapy, and follow-up stool samples were collected after 6 months of treatment. The fecal microbiome, and the proportions of immunoglobulin bound and unbound organisms were determined using flow cytometry. In addition, full-length (long-read) 16S rRNA gene amplicon sequencing technology was used to characterize gut bacteria taxonomies' ASVs at the strain level. Amplicon sequence variants in all samples were evaluated separately for total (presort) bacteria, IgA-coated (IgA+), and IgA-uncoated (IgA–) bacterial fractions. Figure 1 presents an overview of our study design.

**Table:** Demographic and Clinical Characteristics of Study Participants

	Controls	MS (baseline)	MS (6-mo)
Number of participants, n	42	43	19
Age (y), mean ± SD	38.1 ± 11.0	37.6 ± 11.1	39.6 ± 10.2
Sex			
Female, n (%)	31 (73.8)	29 (66.7)	14 (75.0)
Male, n (%)	11 (26.2)	14 (33.3)	5 (25.0)
Race			
Asian, n (%)	3 (7.1)	0 (0.0)	0 (0.0)
Black or African American, n (%)	5 (11.9)	8 (18.6)	5 (26.3)
White, n (%)	29 (69.0)	32 (74.4)	12 (63.2)
More than 1 race, n (%)	2 (4.8)	1 (2.3)	0 (0.0)
Unknown, n (%)	3 (7.1)	2 (4.7)	2 (10.5)
BMI (kg/m^2^), mean ± SD	26.3 ± 5.8	27.2 ± 8.0	27.2 ± 8.5
MS subtype			
Relapsing, n (%)		43 (100)	19 (100)
EDSS, median (range)		2.0 (0–6.5)	2.0 (0–6)
Months since diagnosis, mean ± SD		1.6 ± 4.3	8.7 ± 2.0
Time since relapse			
<30 d, n (%)		8 (18.6)	
30–90 d, n (%)		17 (39.5)	
>90 d, or none recorded, n (%)		18 (41.9)	
High-dose steroid use			
None, n (%)	42 (100)	19 (44.2)	
Within 1 mo, n (%)	0 (0.0)	22 (51.2)	
Within last 2–3 mo, n (%)	0 (0.0)	2 (4.7)	
Anti-CD20 monoclonal, n (%)			
Ocrelizumab			18 (94.7)
Rituximab			1 (5.3)

Abbreviations: BMI = body mass index; MS = multiple sclerosis.

### Gut Microbial α- and β-Diversity Differs in New-Onset Relapsing MS

**Figure 2:**
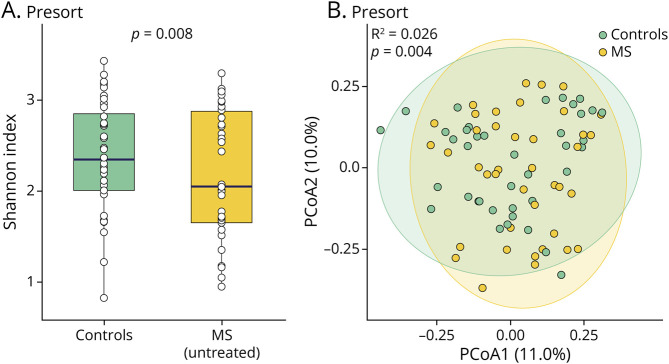
Gut Microbial Diversity in MS Patients at Baseline Compared With Controls (A) Strain-level α-diversity measured by the Shannon Index is shown for total (presort) bacteria. A significant difference in Shannon Index for presort bacteria was observed between the study groups (*p* < 0.05 for the coefficient in a multiple linear regression model adjusted for age, sex, BMI, and steroid use). (B) Strain-level β-diversity, analyzed using Bray-Curtis distance in principal coordinate analysis (PCoA) plots, was compared between patients with MS (gold) and controls (green) for total (presort) bacteria. The percent variance in gut microbiome taxonomic community composition, as explained by PCoA1 and PCoA2, is indicated in their respective axes. R^2^ and *p* values were derived from the adjusted PERMANOVA models. BMI = body mass index; MS = multiple sclerosis; PERMANOVA = Permutational Multivariate Analysis of Variance.

We compared the gut bacterial composition of ASVs between controls and patients with untreated MS. Our evaluation of both strain-level α-diversity (i.e., the microbial diversity within a sample) and β-diversity (i.e., the microbial diversity between groups) found statistically significant differences in both diversity metrics between the total bacteria of patients with MS and that of controls (Figure 2, A and B), in contrast to previous studies.^4,9^ When analyzing IgA+ and IgA– bacterial fractions separately, we did not find significant differences in α- or β-diversity between patients with untreated MS and controls (eFigure 2).

### Host Response to the Gut Microbiome Is Dysregulated in MS

**Figure 3:**
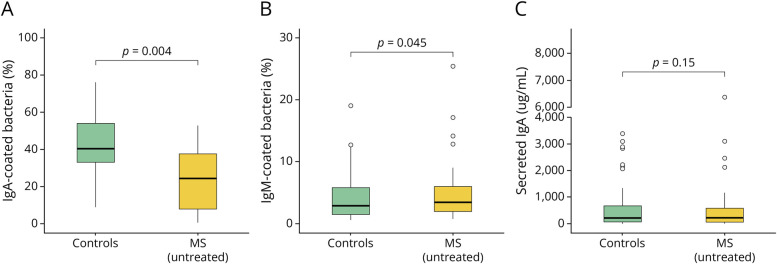
Immunoglobulin Coating in Gut Microbes of Patients With Untreated MS Compared With Controls Flow cytometry was used to determine the proportions of (A) IgA-coated and (B) IgM-coated bacteria. (C) Total secreted, unbound IgA was quantified using ELISA. Multiple linear regression models, adjusted for age, sex, BMI, and steroid use, were applied in (A–C) to assess the significance of differences between controls and patients with untreated MS. BMI = body mass index; IgA = immunoglobulin A; MS = multiple sclerosis.

We used bacterial flow cytometry to quantify the proportion of gut bacteria bound to IgA in patients with untreated MS and controls (eFigure 3). We observed significantly lower proportions of IgA-coated bacteria in patients with untreated MS (Figure 3A, *p* = 0.004). To investigate whether this reduction could be due to a decreased abundance of bacteria that would typically be IgA-coated, we examined whether specific bacterial strains that were IgA-coated in controls could be detected in patients with MS. We identified bacterial strains present in ≥25% of the IgA + fraction samples in the control group and then quantified the relative abundance of these strains in the presort fraction for both controls and untreated MS participants. We did not observe a significant difference in the cumulative sum of relative abundances of these strains between the 2 groups (*p* = 0.79, Mann-Whitney *U* test), nor in the number of these strains present in each sample (*p* = 0.09, Mann-Whitney *U* test) (eFigure 4). Therefore, the decreased proportion of IgA-coated organisms observed in patients with untreated MS compared with controls is not due to the absence of specific bacterial strains in patients with MS. In addition, we observed similar proportions of IgM-coated bacterial strains between controls and patients with untreated MS (Figure 3B, *p* = 0.045), as well as similar levels of secreted, unbound IgA in fecal material between the 2 groups (Figure 3C, *p* = 0.15).

**Figure 4:**
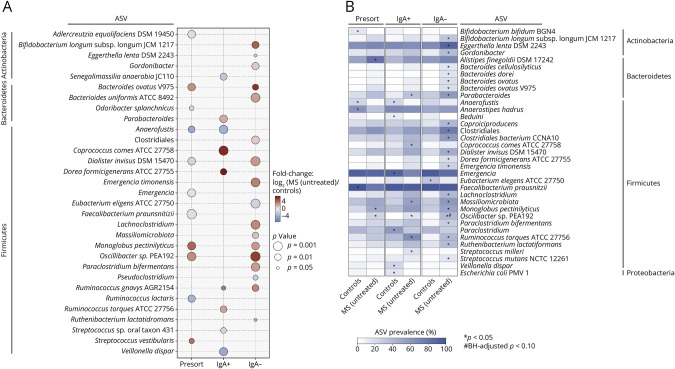
Differential Abundance and Prevalence of Strain-Level ASVs Between Untreated MS and Controls (A) The fold change of ASV relative abundance (calculated as the log ratio of arithmetic means) when comparing untreated MS with controls within the total (presort), IgA+, and IgA– bacterial fractions. Only significant changes are shown in the bubble plot (*p* < 0.05), as identified by the ASV relative abundance coefficient in a multiple linear regression model adjusted for age, sex, BMI, and steroid use. Significant differences are indicated by bubble size, with larger bubbles denoting higher levels of significance; red bubbles represent an increased relative abundance in the untreated MS group, whereas blue indicates a higher abundance in controls. (B) The prevalence of ASVs in each study group across presort bacteria and IgA+ and IgA– fractions. Color saturation in the heatmap indicates ASV prevalence, with darker tones reflecting higher prevalence. * and # indicate statistically significant differences (*p* < 0.05 and Benjamini-Hochberg–adjusted *p* < 0.10, respectively; Fisher exact test). ASV = amplicon sequence variant; BMI = body mass index; IgA = immunoglobulin A; MS = multiple sclerosis.

We further analyzed the composition of the total bacteria and IgA+ and IgA– bacterial fractions for differentially abundant variants between patients with untreated MS compared with controls (Methods). Among the total bacteria, patients with MS displayed decreased relative abundance of a *Faecalibacterium prausnitzii* variant (Figure 4A, *p* = 1.6 × 10^−3^). This anti-inflammatory bacterium was previously observed to be reduced in MS.^4^ By contrast, the novel organism *Monoglobus pectinyliticus* was significantly more abundant in patients with MS (Figure 4A, *p* = 0.017). Within the IgA + fraction, several short-chain fatty acid producers, including *Coprococcus comes* ATCC 27758, *Dorea formicigenerans* ATCC 27755, and 2 *Ruminococcus* variants, were more abundant in patients with untreated MS than in controls. In the IgA– fraction, several variants had higher relative abundances in patients with untreated MS compared with controls, most notably those of *Emergencia timonensis, Oscillibacter*, and *Bifidobacterium longum*. It is interesting, and in contrast to previous studies that reported increased species-level relative abundances of *Akkermansia muciniphila* in patients with MS,^3,24^ that we found no significant differences in the relative abundance of *Akkermansia muciniphila* strain variants between patients with untreated MS and healthy controls.

We also examined the differential prevalence of gut microbial strains, defined as the proportion of individuals for whom a specific organism is present (i.e., above a relative abundance detection threshold of 1.0 × 10^−5^). In the total bacteria, ASVs of *Alistipes finegoldii* DSM17242 and *Monoglobus pectinilyticus* were significantly more prevalent in patients with untreated MS, whereas a *Faecalibacterium prausnitzii* variant was more prevalent in controls (Figure 4B, *p* < 0.05). Distinct prevalence patterns were found among the IgA+ and IgA– fractions. In the IgA + fraction, differentially prevalent ASVs included *Ruminococcus torques* ATCC 27756, a *Massiliomicrobiota* variant, and *C. comes* ATCC 27758.

### B-Cell Depletion Therapy in Patients With MS Results in Altered Gut Microbial Abundance and Prevalence

Repeat stool samples were collected from a subset of patients with MS (n = 19) at 6 months following B-cell–depleting therapy. Clinical follow-up continued for a mean of 30.7 months, and all patients had an excellent clinical response to treatment. No patients experienced clinical relapses after initiating treatment. At the last clinical follow-up, the median Estimated Disability Status Score improved by 1 point in the group of patients who provided 6-month samples. No progression independent of relapse activity could be confirmed (eTable 2).

**Figure 5:**
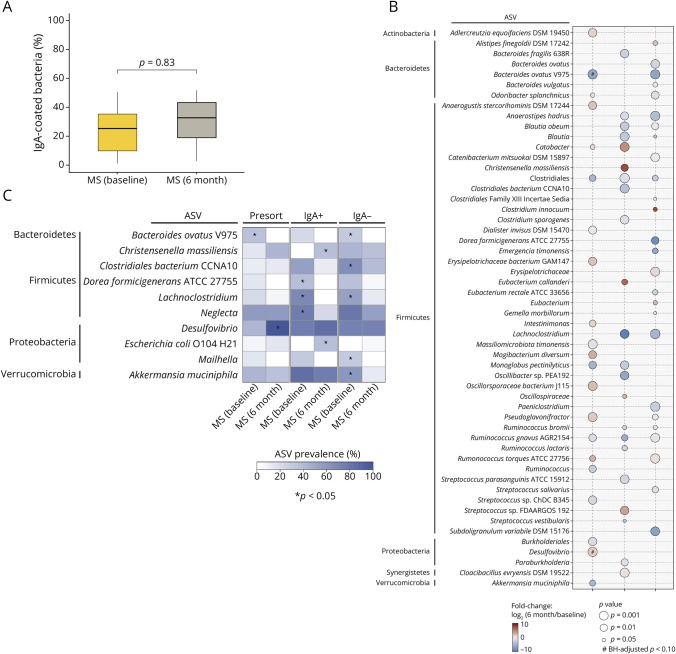
Effect of Anti-CD20 Monoclonal Antibody Treatment on Gut Microbiome in Patients With MS (A) The proportion of IgA-coated bacteria in patients with untreated MS at baseline shows no significant change (*p* = 0.83, mixed-effects linear regression model adjusted for steroid use) after 6 months of treatment. (B) The fold change of strain-level ASV relative abundance from baseline to 6-month posttreatment within the presort, IgA+, and IgA– bacterial fractions. Only significant changes are shown in the bubble plot (*p* < 0.05), as identified by the ASV relative abundance coefficient in a mixed-effects linear regression model adjusted for steroid use. Significant changes in ASVs are indicated by bubble size, with red signifying an increase and blue a decrease posttreatment. # indicates Benjamini-Hochberg–adjusted *p* < 0.10. (C) Differentially prevalent strain-level ASVs between baseline and 6-month posttreatment, with asterisks (*) indicating statistical significance (*p* < 0.05; Fisher exact test). Color saturation reflects the prevalence of ASVs, with darker shades indicating higher prevalence. All statistical comparisons between patients with MS at baseline and 6-month posttreatment were performed on paired samples. ASV = amplicon sequence variant; MS = multiple sclerosis.

There was no significant increase in the proportion of IgA-coated bacteria posttherapy (Figure 5A, *p* = 0.83). In patients with B-cell–depleted MS, a subset of organisms was identified with differential abundance compared with their untreated baseline. Among total bacteria, *Adlercreutzia equolifaciens* DSM 19450 and ASVs of *Mogibacterium diversum*, *Odoribacter splanchnicus*, *Pseudoflavonifractor*, and *Desulfovibrio* showed higher relative abundance at 6-month posttherapy compared with baseline, while *Bacteroides ovatus* V975 was more abundant at baseline (Figure 5B, *p* < 0.05). Several of these variants (including those of *Odoribacter splanchnicus* and *Adlercreutzia equolifaciens*) were previously noted to be increased in controls relative to the untreated MS group (Figure 4A, *p* < 0.05), while other organisms were noticed only after the initiation of B-cell depletion. In the IgA + fraction, an *Lachnoclostridium* variant was notably more abundant at baseline, whereas a *Christensenella massiliensis* variant and several *Streptococcus* variants were significantly more abundant in the posttreatment group (Figure 5B, *p* < 0.05). Finally, in the IgA– fraction, a *Clostridium innocuum* variant showed the highest increase in relative abundance following therapy.

B-cell depletion therapy was associated with significant changes in prevalence, particularly for *Christensenella massiliensis* (present in 31.3% of patients posttherapy vs 0.0% at baseline) and *Akkermansia muciniphila* (5.3% posttherapy vs 52.6% at baseline) variants in the IgA+ and IgA– bacteria, respectively (Figure 5C). Within the total bacteria, a *Desulfovibrio* variant was highly prevalent after B-cell depletion but not at baseline (87.5% posttherapy vs 37.5% at baseline, *p* = 0.01). These results provide insights into the dynamic nature of the host immune response to the gut microbiome and following anti-CD20 treatment.

### New Homeostatic Patterns in Host Immune Response to Gut Microbiome Following Anti-CD20 Monoclonal Antibody Therapy

Immune coating indices indicate the likelihood of bacterial taxa being more frequently found in the IgA + fraction compared with the IgA– fraction.^25,26^ Traditionally, these indices are calculated as the ratio of an organism's average relative abundance in the IgA + fraction compared with its abundance in the IgA– fraction. Most pathobionts exhibit high IgA coating, which likely reflects immune targeting.^25^ Our Immune Coating Score measures the prevalence of a taxon in the IgA + fraction relative to its prevalence in the IgA– fraction (Methods).

**Figure 6:**
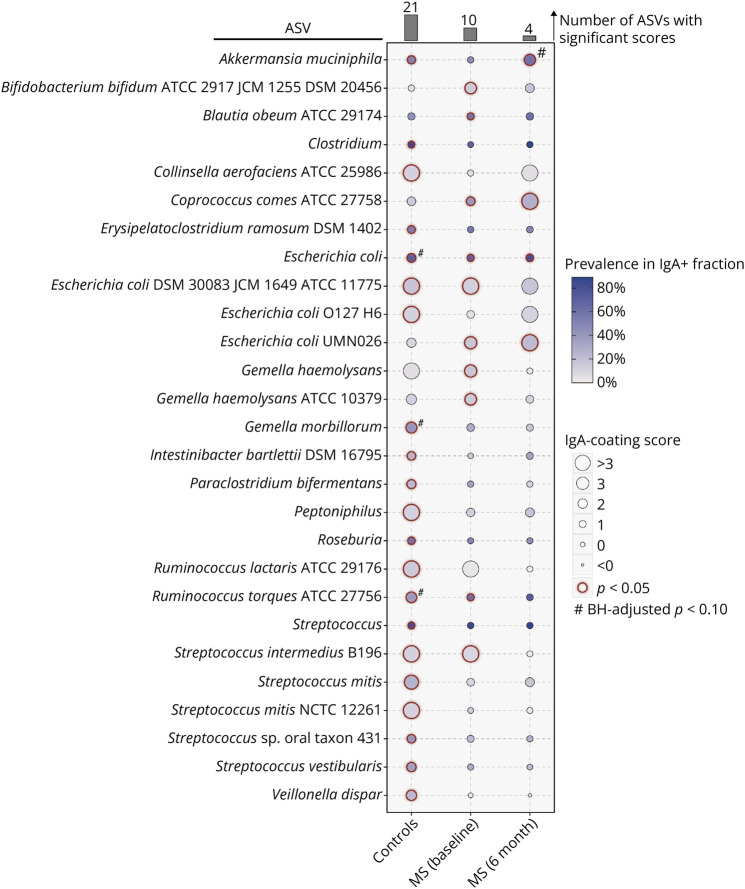
IgA-Coating Patterns of Gut Microbial Strains Across Study Groups The IgA-Coating Scores (ICSs) for strain-level ASVs are shown in the bubble plot for controls, patients with untreated MS at baseline, and patients with MS 6-month posttreatment. The size of the bubbles reflects the ICS, with larger bubbles indicating a higher degree of IgA binding to ASVs. The color saturation of the bubbles corresponds to the prevalence of each ASV within IgA + samples, with darker shades indicating higher prevalence. ASVs with a statistically significant ICS (*p* < 0.05, permutation test) are outlined in dark red. # indicates Benjamini-Hochberg–adjusted *p* < 0.10. ASV = amplicon sequence variant; MS = multiple sclerosis.

Using our Immune Coating Score approach to identify bacterial strains more likely to be IgA-coated than uncoated (Methods), we found 21 ASVs with significant scores (*p* < 0.05, permutation test) among controls, with variants of *Collinsella aerofaciens*, *Escherichia coli*, and *Streptococcus mitis* showing the highest scores (Figure. 6). By contrast, patients with untreated MS had only 10 variants with significant Immune Coating Scores. After 6 months of B-cell depletion therapy, we observed that certain organisms (*Akkermansia muciniphila*, *Bifidobacterium bifidum*, *Blautia obeum*, and *Gemella*) displayed immune coating patterns more similar to those of controls than to patients with untreated MS. Other variants developed IgA-coating patterns that were different from those observed in either controls or untreated MS, as seen with the increased Immune Coating Score of *Coprococcus comes* ATCC 27758 and *Escherichia coli* UMN026, and the loss of IgA coating in *Ruminococcus torques* ATCC 27756 and *Streptococcus intermedius* B196. Overall, these results suggest key differences in the immune response to the gut microbiome between healthy individuals and patients with MS, both before and after 6 months of B-cell depletion therapy.

## Discussion

Although gut microbiome dysbiosis is now recognized in MS, the interactions between the gut microbiome and the host's immune response to this disease remain largely unexplored. In this study, we focused on a cohort of patients with newly diagnosed, untreated MS using a long-read, ASV detection technique to achieve strain-level taxonomic resolution. We examined the host's immunologic response—specifically, IgA-binding patterns—to the gut microbiome following anti-CD20 B-cell depletion therapy. New-onset, untreated patients with MS show significant reductions in IgA-bound fecal microbiota, as well as changes in the abundance and prevalence of specific gut bacterial strains within both the total and IgA-bound bacterial fractions. Immune Coating Scores identified specific organisms where IgA-coating patterns were lost following B-cell–depleting therapy, as well as organisms whose IgA-coating patterns shifted to align more closely with controls. This strain-level analysis using ASVs elucidates shifts in gut microbial taxa induced by immune perturbation in MS suggesting the potential for leveraging these changes as biomarkers or therapeutic targets.

Although most previous studies did not find significant differences in α- and β-diversity of the gut microbiome between patients with MS and controls,^9,27^ we observed significant differences in both diversity metrics among the total bacteria of patients with MS compared with controls. Others have observed trends for reduced diversity among individuals with MS, with a few more clinically homogeneous cohorts reaching statistical significance.^28^ We observed no differences in α- and β-diversity among IgA+ and IgA– bacterial fractions, suggesting that microbial diversity remains stable despite changes in immune coatings to gut bacteria. In addition, we identified differential abundances of specific organisms, including a *Faecalibacterium prausnitzii* variant, which has previously been reported to have reduced relative abundance in patients with newly diagnosed, untreated MS.^4,9^ *Faecalibacterium prausnitzii* is a major producer of butyrate, an immunomodulatory short-chain fatty acid that has been shown to ameliorate symptoms in a mouse model of MS.^29,30^ Gut microbial-derived short-chain fatty acids have been reported to influence the immune system in a variety of ways, including enhancing regulatory T-cell function and regulating NF-kB signaling in macrophages and dendritic cells.^31^ They were also found to be critical for maintaining normal microglial maturation and function.^32^ A decline in short-chain fatty acid-producing bacteria is a compelling hypothesis for how the gut microbiome may interact with autoimmune diseases such as MS.^5,33,34^

Disruption of intestinal barrier function, a phenomenon previously reported in patients with MS,^35–38^ is another mechanism through which specific intestinal bacteria may affect MS pathology. Gut barrier integrity can be maintained by short-chain fatty acids^34^ and is supported by mucin (secreted by goblet cells) and pectin (obtained through diet).^39^ Disrupting bacteria that produce or metabolize short-chain fatty acids, mucin or pectin could potentially affect the gut epithelial barrier. It is interesting that in our cohort of patients with newly diagnosed MS, we observed enrichment of *Monoglobus pectinilyticus,* a recently discovered pectin-degrading organism.^40^ This observation complements other studies that have reported an elevation of the mucin degrader *Akkermansia muciniphila* in MS.^4,9^ Future studies will be needed to determine whether dysregulation of such microbes contributes to the “leaky gut” phenomenon observed in MS.^35,41^

The specific organisms identified in this study overlap partially with those identified by others.^9,27^ Differences between studies are not surprising, given methodological differences in sequencing and analysis, as well as geographic differences in the populations recruited. Our use of long-read 16S rRNA gene amplicon sequencing offers improved taxonomic resolution compared with short-read sequencing, which was used by many previous MS studies. This key difference may have allowed the identification of changes in specific strains that could not have been observed when limited to comparing higher taxonomic levels.

Our work illustrates that the host response to the gut microbiome, including relationships between IgA+ and total gut bacteria, is dysregulated in early-stage MS, building on previous work where decreased proportions of IgA + gut bacteria were associated with high levels of physical disability and recent clinical relapses.^18,42^ We observed a significantly reduced proportion of IgA-coated gut bacteria at the onset of clinical MS despite similar levels of secreted fecal IgA between patients with MS and controls. This implies that IgA-binding affinity could be fundamentally compromised in MS, perhaps through B-cell dysfunction, posttranslational modifications that reduce antibody polyreactivity, deglycosylation of secretory immunoglobulin A by *Akkermansia muciniphila*, or other factors that inhibit polyreactive antibody binding.^43,44^

IgA and IgA-secreting cells are increasingly recognized for their potential significance in MS. Studies have shown that microbial-reactive, IgA-producing plasma cells migrate from the gut to the CNS during active MS flares,^17,18^ and there have been reports of cerebrospinal fluid (CSF)-restricted IgA synthesis in the early stages of MS.^45^ Our data suggest that in patients with MS, IgA-secreting lymphocytes within the gut mucosa may generate antibodies with impaired antigen recognition, potentially contributing to microbial dysregulation. Although IgA-secreting cells derive from the B-cell lineage, anti-CD20 antibodies incompletely deplete gut-resident plasma cells and circulating IgA + plasmablasts.^46,47^ A single previous study on the relationship between ocrelizumab treatment and the gut microbiome found no significant changes in α- or β-diversity with treatment, and reported only small changes, mainly at the phylum level, in specific microbes.^48^ To date, no studies have examined the effect of B-cell depletion on gut microbe-IgA–coating relationships in MS. We postulated that B-cell–depleting medications would not significantly affect the gut microbiome and IgA-coating patterns. In contrast to this, our study observed that B-cell depletion correlated with shifts in the differential abundance and prevalence of certain organisms among total, IgA+ and IgA– bacterial subsets without significantly altering the total proportion of IgA-bound microbes. For example, ASVs for *Mogibacterium diversum*, *Odoribacter splanchnicus*, *Pseudoflavonifractor*, *and Desulfovibrio* had higher relative abundance after 6 months of B-cell depletion, and a *Desulfovibrio* variant became more prevalent in the presort bacteria group after treatment. Furthermore, the number of strains with significant IgA-coating scores decreased in patients with untreated MS compared with controls. However, we observed a posttreatment increase in IgA-coating scores for specific gut microbes (*Coprococcus comes* ATCC 27758 and *Escherichia coli* UMN026) and a re-emergence of a significant score for *Akkermansia muciniphila*. These findings suggest that anti-CD20 therapy influences the homeostasis between IgA and gut microbes in a strain-specific manner.

Although our study represents an advancement in the field with its clinically uniform cohort, longitudinal sampling, integration of IgA binding, and use of long-read amplicon sequencing for strain-level detection, we acknowledge several methodological limitations. First, owing to the observational nature of this study, it remains unclear which of the identified strains are directly involved in disease processes or are clinically relevant. Elucidating the pathogenic implications of changes in the host immune response to gut microbes following B-cell depletion therapy is a priority for future research. Second, the strains we identified as differentially abundant differ from those in previous studies,^9,27^ a discrepancy commonly seen in microbiome research that may stem from geographic, lifestyle, or phenotypic differences among participants, as well as variations in sample sizes or analytical methods used. However, since ASVs use exact sequences for more precise microbial identification,^49^ our results can be easily compared with future studies that sequence the full length of 16S rRNA genes. Future studies should include multiple sites and larger cohorts to enhance the generalizability of our findings. In addition, the computational tools appropriate for microbiome analysis are still developing, and the selection of methods for statistical hypothesis testing can substantially influence study outcomes.^50^ Third, we used magnetic sorting to separate IgA-coated bacteria from uncoated bacteria. Since flow cytometry-based cell sorting could lead to a higher level of purity compared with magnetic sorting, this methodology could be considered for future studies. Fourth, we chose to present uncorrected significance because most differences were undetectable after applying the Benjamini-Hochberg false discovery rate correction. This may reflect the absence of stark differences in individual strains between study groups, the modest size of our exploratory pilot study, the high dimensionality of the strain-level ASVs tested, and the lack of independence among individual tests (a key assumption in the Bonferroni and Benjamini-Hochberg procedures). Although multiple hypothesis correction is essential for reducing false positives, we opted to prioritize the detection of true differentially abundant variants, which could have been missed with strict correction. Instead, we carefully selected our cohort and accounted for fixed demographic variables (age, sex, BMI, and steroid use) in our statistical models. Finally, future investigations should explore the ecological interactions within microbial communities in health and disease contexts, recognizing that the pathogenic potential in MS may not lie within individual organisms in isolation, but rather within networks of microbes occupying critical biological niches.^51^

As we refine our understanding of the gut microbiome's role in MS, there are multiple potential opportunities for clinical translation. A deeper study of disease-associated commensals, some of which exhibit molecular mimicry with CNS autoantigens,^52,53^ may improve our understanding of MS pathogenesis and the immune mechanisms that drive the disease. Expanding our knowledge of disease-associated microbial patterns could lead to clinical applications, such as using gut microbiome features as diagnostic or prognostic biomarkers. This may improve our ability to detect early, or even preclinical, stages of MS. Finally, animal models suggest that directly modulating the gut microbiota could have therapeutic benefits^17^; but it remains to be seen whether strategies such as prebiotics, probiotics, or fecal microbial transplants will prove effective in the more complex clinical setting of MS.

## Glossary

ASV: amplicon sequence variant
BMI: body mass index
GDP: guanosine diphosphate
ICS: IgA-Coating Score
IgA: immunoglobulin A
MLRM: multiple linear regression model
MS: multiple sclerosis
